# A Novel Self-Assembly Strategy for the Fabrication of Nano-Hybrid Satellite Materials with Plasmonically Enhanced Catalytic Activity

**DOI:** 10.3390/nano11061580

**Published:** 2021-06-16

**Authors:** Gareth Morris, Ioritz Sorzabal-Bellido, Matthew Bilton, Karl Dawson, Fiona McBride, Rasmita Raval, Frank Jäckel, Yuri A. Diaz Fernandez

**Affiliations:** 1 Surface Science Research Centre, Department of Chemistry, University of Liverpool, Liverpool L69 3BX, UK; G.Morris@liverpool.ac.uk (G.M.); ioritz.sorzabal-bellido@liverpool.ac.uk (I.S.-B.); fiona.mcbride@liverpool.ac.uk (F.M.); 2 Stephenson Institute of Renewable Energy and Department of Physics, University of Liverpool, Liverpool L69 3BX, UK; 3 Albert Crewe Centre for Electron Microscopy, University of Liverpool, Liverpool L69 3BX, UK; M.W.Bilton@liverpool.ac.uk; 4 Department of Mechanical, Materials and Aerospace Engineering, School of Engineering, University of Liverpool, Liverpool L69 3BX, UK; K.Dawson@liverpool.ac.uk

**Keywords:** plasmonic material, plasmon enhanced catalysis, silver nanoparticles, quantum dots, water splitting, hydrogen generation, renewable energy

## Abstract

The generation of hydrogen from water using light is currently one of the most promising alternative energy sources for humankind but faces significant barriers for large-scale applications due to the low efficiency of existing photo-catalysts. In this work we propose a new route to fabricate nano-hybrid materials able to deliver enhanced photo-catalytic hydrogen evolution, combining within the same nanostructure, a plasmonic antenna nanoparticle and semiconductor quantum dots (QDs). For each stage of our fabrication process we probed the chemical composition of the materials with nanometric spatial resolution, allowing us to demonstrate that the final product is composed of a silver nanoparticle (AgNP) plasmonic core, surrounded by satellite Pt decorated CdS QDs (CdS@Pt), separated by a spacer layer of SiO_2_ with well-controlled thickness. This new type of photoactive nanomaterial is capable of generating hydrogen when irradiated with visible light, displaying efficiencies 300% higher than the constituting photo-active components. This work may open new avenues for the development of cleaner and more efficient energy sources based on photo-activated hydrogen generation.

## 1. Introduction

One of the grand challenges in nanotechnology is the design and fabrication of complex materials able to deliver enhanced performances in terms of catalytic activity. Even a marginal increase in reaction yield aided by catalysis can have a huge economic and environmental impact within large-scale industrial settings [1]. This is particularly relevant for the energy sector, where many alternative energy sources [2], such as photo-activated hydrogen generation [3,4], are facing huge translational barriers due to the relatively low efficiency of current photo-catalysts.

Considerable research effort has been devoted to improve the photo-catalytic efficiency of existing semiconductor materials by applying top down and bottom up nano-engineering approaches [5]. Major improvements have been achieved by increasing the complexity of the materials at the nanoscale, favouring local charge separation and directing the systems towards specific reaction pathways [6,7]. One area of particular interest is plasmon enhanced catalysis, where only a few examples relevant to hydrogen evolution photo-chemistry have been explored [8,9,10]. Research into this field is so challenging that even an increase of 50% in catalytic activity is a significant achievement [10]. Several mechanisms have been proposed to explain the enhanced photocatalytic activity observed in different plasmonic systems, including photo-thermal effects [11,12], energy transfer [13], and field enhancement [14].

**Figure 1:**
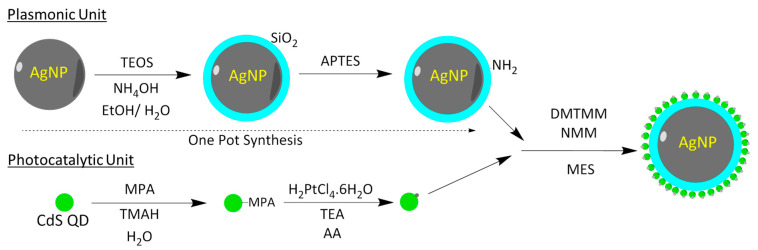
Strategy for the fabrication of nano-hybrid satellite material based on AgNPs and CdS quantum dots. Experimental details of the synthesis are presented in the Methods section. Acronyms: TEOS: Tetraethyl orthosilicate. APTES: (3-aminopropyl)triethoxysilane. MPA: 3-mercaptopropionic acid. TMAH: Tetramethylammonium Hyroxide. TEA: Triethanolamine. AA: L-Ascorbic acid. DMTMM: 4-(4,6-dimethoxy-1,3,5-triazin-2-yl)-4-methylmorpholinium chloride. NMM: 4-methylmorpholine. MES:2-(4-morpholinol)ethanesulfonic acid.

In this paper we present a new type of nano-hybrid material (Figure 1), obtained by applying the modular principles of supramolecular chemistry [15,16]. Experimental data showed the intrinsically complex nano-scale structure of the materials obtained, resembling a satellite system of photo-active particles surrounding a bigger plasmonic-antenna core. We observed an unprecedented 300% increase in the efficiency of photo-activated hydrogen generation, paving the way for new applications in this field.

## 2. Results and Discussion

### 2.1. Fabrication Strategy for the Nano-Hybrid Satellite Materials

To overcome the challenge of creating a complex nanomaterial, incorporating both plasmonic and photo-catalytic properties within the same nano-structure, we propose a multi-step self-assembly strategy described in Figure 1, following modular approaches inspired by supramolecular chemistry [15,16]. CdS quantum dots (QDs) decorated with platinum clusters were created, since they are known to catalyse hydrogen generation reactions via photo-activated water splitting. Spherical CdS@Pt QDs have been extensively investigated before [17,18,19,20] and constitute a good model system to be used within more complex settings, such as the nano-hybrid materials investigated here. The QDs were prepared in several steps, starting from an organic phase, they were functionalised with 3-mercaptopropionic acid and then transferred into an aqueous phase for the photo-deposition of Pt clusters at the surface of CdS QDs (Figure 1, further details in the Materials and Methods section).

In parallel, we synthesised quasi-spherical silver nanoparticles (AgNPs), which constituted the plasmonic core of the satellite material. Subsequently, these AgNPs were covered with a thin coating of silica, functionalised at the surface with amino groups by the incorporation of (3-aminopropyl)triethoxy silane (APTES). The thickness of the SiO_2_ layer was accurately controlled by varying the conditions of the synthesis, as described in the following sections. This SiO_2_-NH_2_ layer acted as spacer between the plasmonic core and the satellite QDs. The presence of amino groups at the AgNP@SiO_2_ surface allowed the formation of covalent bonds with the carboxylate moieties at the surface of the QDs, via peptide-like coupling, generating a nano-hybrid material with a plasmonic AgNP core surrounded by satellite photo-catalytic CdS@Pt QDs. In the following sections we will demonstrate how the individual properties of each functional component were preserved along the multi-step fabrication process, allowing the investigation of the photo-catalytic response for this nano-hybrid satellite system.

### 2.2. The Photo-Catalytic Unit: CdS QDs Functionalised with Pt Clusters

**Figure 2:**
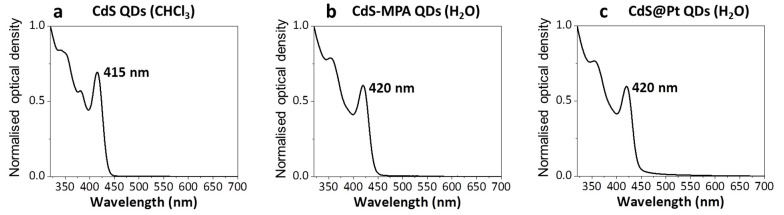
Representative UV-Vis spectra of different steps of the CdS QDs preparation: (**a**) Initial dispersion in chloroform of CdS QDs as synthesised. (**b**) CdS QDs phase transferred into aqueous phase. (**c**) Photo-deposition of Pt clusters on CdS QDs. All spectra are normalised for comparison.

The process for the synthesis of the CdS@Pt QDs encompassed several steps. Initially [14,15,16], the CdS QDs were obtained from the reaction of cadmium oleate with elemental sulfur in 1-octadecane, under inert gas conditions, followed by repeated purification steps of centrifugation and re-dispersion, until a strong-yellow colloidal solution of CdS QDs in chloroform was obtained. The typical UV-Vis extinction spectra of mono-disperse CdS QDs displayed a very sharp 1st exciton peak in the visible region and higher order peaks in the UV [21] (Figure 2a). For non-coordinating solvents such as chloroform, the wavelength of the 1st exciton peak (λ) can be related to the diameter (*D*) of the CdS QDs by the empirical Equation (1) [20]:

(1) $$D=\left(-6.6521\times 10^{-8}\right)\lambda^{3}+\left(1.9557\times 10^{-4}\right)\lambda^{2}-\left(9.2352\times 10^{-2}\right)\lambda -(13.29)$$

**Figure 3:**
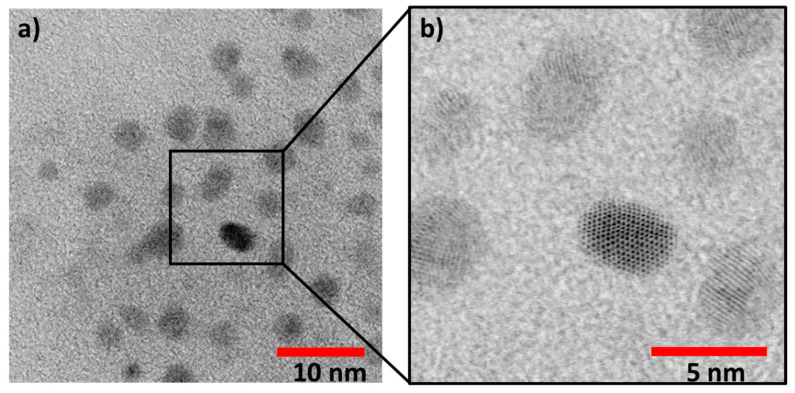
Bright-Field Scanning Transmission Electron Microscopy (BF-STEM) images of the CdS@Pt QDs: (**a**) Representative BF-STEM image of CdS@Pt QDs. (**b**) Higher magnification BF-STEM of a selected area.

Using this expression and the position of the peak at 415 nm in the UV-Vis spectrum in chloroform (Figure 2a), we calculated a diameter of 3.9 nm for the CdS QDs. This value was consistent with the particle size of 3.8 ± 0.4 nm, determined by using Bright-Field Scanning Transmission Electron Microscopy (BF-STEM) (Figure 3).

The CdS QDs were subsequently transferred into an aqueous phase, after functionalisation with 3-mercaptopropionoic acid (MPA). We observed a small red shift of the maxima from 415 nm for the QDs in chloroform, to 420 nm in the aqueous phase, probably due to changes in the coordination environment of the QDs by the presence of strongly binding MPA molecules and the transfer into a more polar medium (Figure 2b). The extinction peak was still narrow, indicating the presence of mono-disperse QDs after the phase transfer from chloroform into the aqueous phase. Finally, the photo-deposition of platinum clusters on the QDs was performed, following a method previously reported [18,19]. The incorporation of the Pt clusters onto the CdS QDs did not modify the optical properties of the material, showing the maxima of the extinction spectrum at 420 nm (Figure 2c). These CdS@Pt QDs were then purified from excess of reagents by centrifugal ultrafiltration, leading to a material that was stable in the dark for weeks under air and refrigerated conditions. This material showed good photo-catalytic response for hydrogen generation from water, displaying reaction rates as high as 40.4 mol H_2_/min produced per mol of Pt in the reaction solution under our experimental conditions (see Materials and Methods section for details). We observed some batch-dependent variability on the H_2_ evolution reaction rates, mainly due to heterogeneities on the platinum photo-deposition step, which are well documented in the literature [18]. For this reason, we used a unique batch of CdS@Pt QDs for all the experiments described in the following sections.

### 2.3. Plasmonic Antenna: Quasi-Spherical Silver Nanoparticles Coated with Amino Functionalised SiO_2_ Spacing Layer (AgNP@SiO_2_-NH_2_)

**Figure 4:**
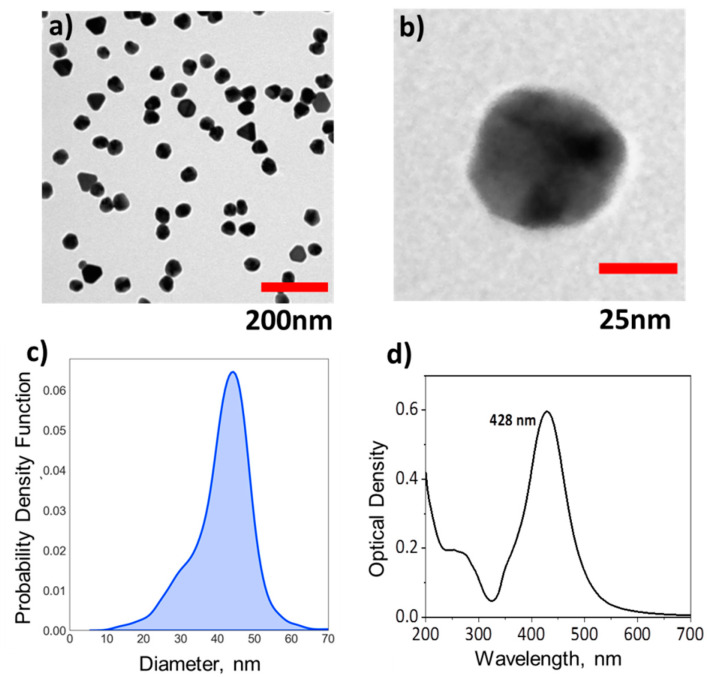
Plasmonic AgNPs as synthetised: (**a**,**b**) Representative TEM images of AgNPs at different magnifications. (**c**) Size distribution of AgNPs. (**d**) Extinction spectra of AgNPs showing the characteristic plasmonic peak at 428 nm.

The preparation of the plasmonic units started with the synthesis of quasi-spherical silver nanoparticles (AgNPs), using a combination of silver nitrate, tannic acid and sodium citrate [22]. This reaction led to a clear-yellow colloidal solution, with the characteristic plasmonic band at 428 nm for the AgNPs (Figure 4d). Despite the presence of a few atypical particles with non-spherical shapes, the population was mono-disperse in size, showing a relatively narrow size distribution centred at 42 nm (Figure 4). The synthesis was reproducible and the AgNPs obtained after purification by centrifugation and re-dispersion in sodium citrate, were stable at room temperature for several weeks.

**Figure 5:**
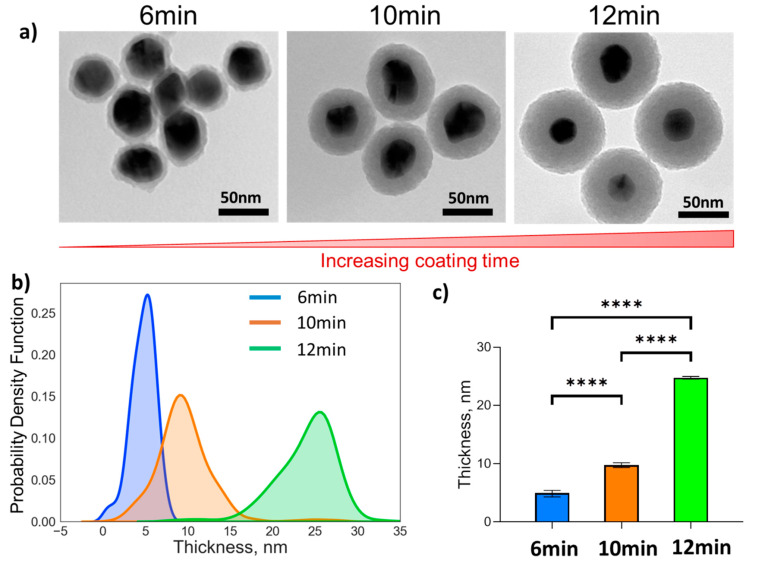
Silica-coated AgNPs: (**a**) Representative TEM images of AgNP@SiO_2_-NH_2_ for different reaction times (6, 10 and 12 min of SiO_2_ coating followed by 4 min APTES functionalisation). (**b**) Population distributions of the thickness of the silica layer on AgNP@SiO_2_-NH_2_ for different reaction times as determined from semi-automated analysis of TEM images. (**c**) Bar chart showing average thickness of the silica layer on AgNP@SiO_2_-NH_2_ with 95% confidence interval error bars. **** indicates a significant difference between groups (*p* < 0.0001).

Subsequently, the AgNPs were coated with a layer of SiO_2_ using tetraethyl orthosilicate (TEOS) under basic conditions [23,24]. This simple, yet robust process allowed direct control of the thickness of the SiO_2_ layer by changing the reaction time. The different coatings were directly analysed by TEM (Figure 5a), exploiting the contrast in electron density between the silver core and the SiO_2_ layer, that allowed the implementation of a semi-automated image segmentation workflow for processing a large number of images for each experimental condition (Supplementary Materials Figures S1 and S2 and Table S1). Using this analysis method, we observed a non-linear relationship between reaction time and thickness of the SiO_2_ layer, but fine control over reaction time was sufficient to obtain AgNP@SiO_2_ with coatings varying from a few nm to tens of nm. Interestingly, the dispersity of the thickness distribution grew larger for longer reaction times (Figure 5b), however the differences between different coating conditions lead to statistically distinct materials (Figure 5c).

We then exploited the reactivity of the SiO_2_ coating to introduce (3-aminopropyl) triethoxysilane (APTES) molecules, bearing primary amino groups that could serve as anchoring points for the CdS@Pt QDs functionalised with carboxylate moieties from MPA ligands. The reaction time for APTES was kept constant for the different SiO_2_ coatings, allowing control over the thickness of the final coating by a single parameter, namely TEOS reaction time, as discussed above. The successful functionalisation with APTES was demonstrated by XPS analysis, showing the presence of nitrogen on the AgNP@SiO_2_-NH_2_ material (Supplementary Materials Figure S3).

### 2.4. Nano-Hybrid Satellite Material Combining AgNP@SiO_2_-NH_2_ Plasmonic Antenna and Photo-Catalytic CdS@Pt QDs

In the previous sections we described the fabrication of the key modular components of our nano-hybrid material, namely the plasmonic-core antenna AgNP@SiO_2_-NH_2_ and the photocatalytic unit CdS@Pt QDs. The final step of the synthesis process required the self-assembly and stabilisation of the satellite system. Within the design strategy, we envisioned that the formation of covalent bonds between the primary amino groups on the AgNP@SiO_2_-NH_2_ core and the carboxylate moieties on CdS@Pt QDs functionalised with MPA could be achieved by the use of the amide coupling reaction, activating the carboxylate of MPA with DMTMM. This reaction has been extensively used for the synthesis of peptides from amino acid precursors, combining, like in our case, primary amino groups and carboxylates to form amide bonds in aqueous media [25,26,27]. Controlling the pH of the solution is critical for this coupling reaction, and we ensured an optimal pH = 5.5 by using MES buffer (further details can be found in the Materials and Methods section). After the reaction was completed, the excess of coupling agents could be easily removed by centrifugation and re-dispersion of the assembled nanoparticles in water.

**Figure 6:**
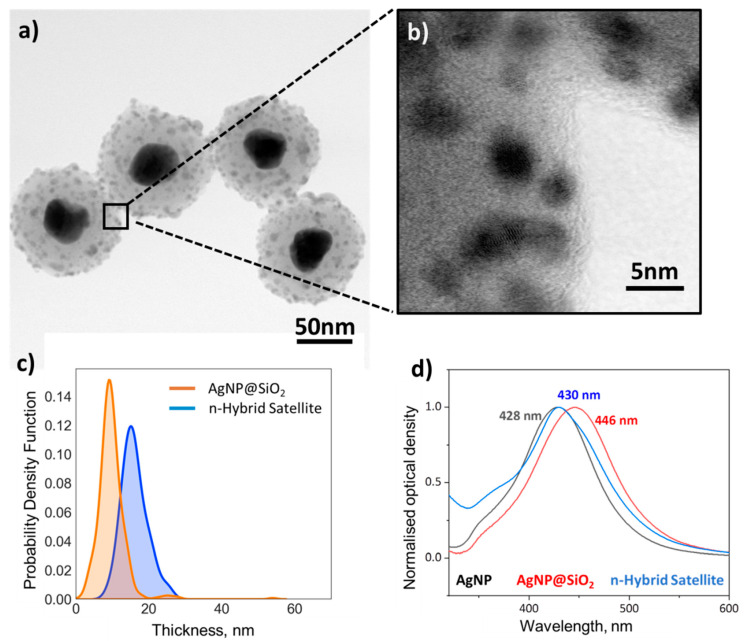
Nano-hybrid satellite material: (**a**) Representative BF-STEM image of the nano-hybrid satellite materials showing the AgNPs plasmonic antenna surrounded by the SiO_2_ spacing layer and the photo-catalytic CdS@Pt QDs. (**b**) Higher magnification BF-STEM image of a selected area showing the CdS@Pt QDs on the outer SiO_2_ layer; (**c**) Population distribution of the thickness of the coating before and after incorporation of the QDs; Difference on SiO_2_ outer layer thickness before and after incorporation of the QDs was 6.3 nm (±5.3 nm). (**d**) Representative normalised optical densities for silver nanoparticles (AgNPs, black), silver nanoparticles coated with SiO_2_ (AgNP@SiO_2_, red), and the nano-hybrid satellite nanomaterial (blue).

STEM images of this hybrid nano-material showed the presence of the CdS@Pt QDs on the SiO_2_ outer layer around the core plasmonic AgNPs, where the QDs were visible as dark areas of higher electron density (Figure 6a,b). Interestingly, some of the QDs appeared imbedded within the SiO_2_ layer, but this may be due to the intrinsic limitation of STEM, showing only a 2D projection of the QDs at the front and back of the cross-section. The thickness of the outer silica layer remained similar before and after incorporation of the CdS@Pt QDs (Figure 6c and Supplementary Materials Table S1), as did the size of the QDs compared to the precursor nanomaterials discussed in the previous sections (Figure 6b).

Interestingly, the optical properties of the nano-hybrid system cannot be simply explained by a linear combination of the spectra of the two precursor materials. The initial position of the spectral maximum for the CdS@Pt QDs is at 420 nm, while the characteristic plasmonic peak for AgNPs red shifted from 428 nm to 446 nm by the incorporation of the SiO_2_ coating. Subsequent assembly of AgNP@SiO_2_-NH_2_ and CdS@Pt QDs placed the maximum wavelength for the nano-hybrid system at 430 nm (Figure 6d). In relative terms, if we were to assume that the spectral properties of the two components were additive upon assembly, their individual contributions to the total extinction spectrum could be estimated considering the regions of the spectrum where only one of the two components shows high optical density, i.e., above 450 nm only AgNP@SiO_2_-NH_2_ contributes to the optical density, while below 350 nm the optical properties are dominated by CdS@Pt QDs (Supplementary Materials Figure S4). However, any attempt to reconstruct the nano-hybrid satellite spectra by a linear combination of these two components failed, suggesting that the optical properties of the resulting material are not the simple superposition of the individual constituents. Nonetheless, the considerable overlap of the UV-visible spectra for the core AgNPs and the satellite CdS@Pt QDs is particularly promising in terms of the effects it may bring to the photo-catalytic response of the nano-hybrid system, investigated in the following section.

**Figure 7:**
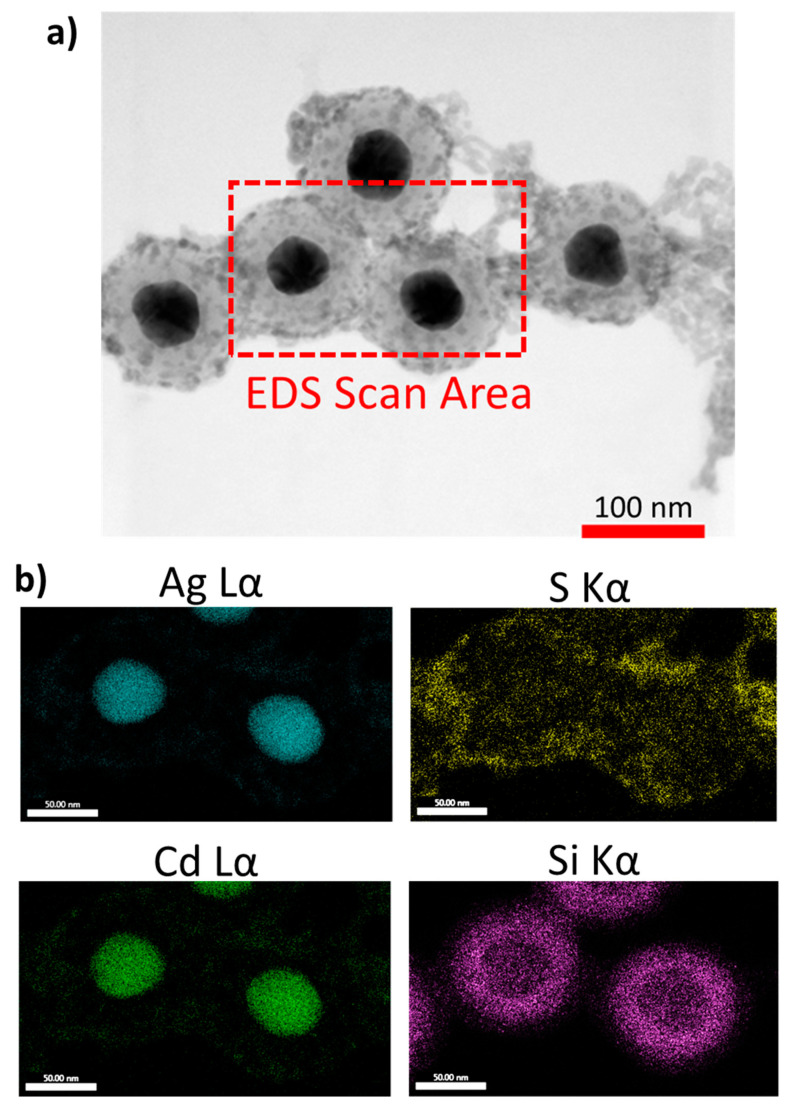
Chemical composition of the nano-hybrid satellite material: (**a**) Representative BF-STEM image of nano-hybrid satellite materials. (**b**) EDS map of a selected area from frame A showing the distribution of different elements. EDS data for Cd and Ag must be interpreted with caution due to energy overlap (see Supplementary Materials Figures S6–8 and Table S3 for further details).

The successful fabrication of the nano-hybrid satellite system was also probed by energy dispersive X-ray spectroscopy (EDS). This technique combines the enhanced resolution imaging capability of STEM with localised chemical analysis. The presence of the AgNP core, the silica spacing layer and the CdS@Pt QDs satellites were clearly observed in the STEM-EDS images (Figure 7). Despite the system limitations to separate the EDS spatial distributions for Ag and Cd due to energy overlap and the low concentrations of Pt in the sample, which is below detection limit for EDS, by analysing the distribution of sulfur coming from CdS and located in the outer shell of the structures, we inferred the localisation of CdS@Pt QDs in the outer SiO_2_ layer of the hybrid satellite nanomaterials. The presence of Pt, Cd, Ag, S and Si in the final product was also confirmed by XPS analysis (Supplementary Materials Figure S5), however the limited spatial resolution of this technique (i.e., ≈1 µm in XY) precluded more detailed analysis of the location of these elements at the nanoscale.

### 2.5. Photo-Activated Hydrogen Generation by Nano-Hybrid Satellite Materials

**Figure 8:**
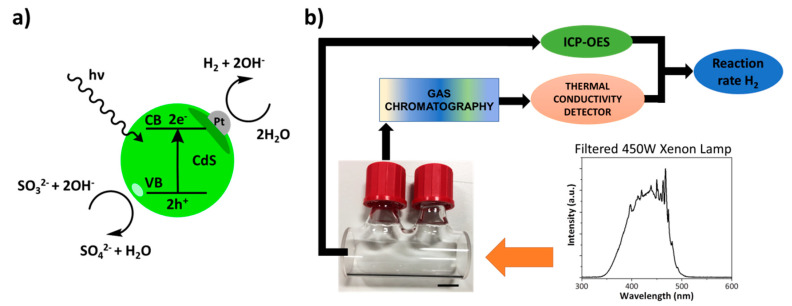
Photo-activated hydrogen evolution: (**a**) Schematic representation of the reaction on CdS@Pt QDs [28]; (**b**) Diagram of the experimental approach used to evaluate the hydrogen evolution reaction rates. The photograph inset shows the 5 cm long quartz cell used as the reaction chamber.

Finally, we investigated the photocatalytic activity of the nano-hybrid satellite material fabricated and characterised in the previous sections. For these experiments, the photo-activated hydrogen-evolution was chosen as a model reaction that has been extensively studied for freely dispersed CdS@Pt QDs before. At the microscopic level, this reaction involves the formation of electron-hole pairs within the semiconductor CdS QDs, induced by the incident light [4,28]. These electron-holes are separated, with the electrons transferring to the Pt clusters at the surface of the QDs, followed by two half-reactions leading to the completion of the redox cycle (Figure 8A). The Pt clusters catalyse the reduction of water by electrons to release hydrogen, while the holes are generally removed by a hole scavenger, since otherwise the redox cycle cannot be completed and the QDs degenerate with time. Na_2_SO_3_ has been extensively used as a hole-scavenger for this type of photo-activated reactions [29], and therefore it was the first choice for our reaction system.

Our experimental set up (Figure 8b) consisted of a 5 cm long cylindrical reaction cell made of quartz with two gas-tight caps, exposed to a 450 W Xenon lamp. The light of the lamp was filtered to deliver a relatively flat intensity profile in the spectral region between 350 nm and 480 nm. The reaction cell was partially filled up to 38% of the volume with a deaerated solution containing the photo-active particles in solution (either CdS@Pt QDs or the satellite material dispersions) and the hole-scavenger (Na_2_SO_3_ in solution), allowing the rest of the space within the cell to be occupied by an inert carrier gas (i.e., high purity Argon at 1 atm). The generation of hydrogen within the photo-reaction cell was accurately determined by gas chromatography coupled to a thermal conductivity detector, after taking gas aliquots from the headspace of the reaction cell. To allow comparison between the free QDs and the nano-hybrid satellites, we used for the control experiments the same batch of CdS@Pt QDs that were used to fabricate each specific satellite system. Additionally, we normalised the moles of hydrogen produced by considering the moles of Pt present in each solution, determined independently by ICP-OES (data shown in Supplementary Materials Table S2). This normalisation allowed us to express the hydrogen evolution data in terms of reaction rates that can be directly compared, irrespective of the actual concentration of catalyst present in each solution.

The results, presented in Table 1 for a representative satellite system (i.e., SiO_2_ layer 13.1 nm ± 1.6 nm), showed that both the free QDs and the nano-hybrid satellite material were able to generate hydrogen under these experimental conditions. Remarkably, the hydrogen evolution rates for the nano-hybrid satellites were higher than the rates for the free CdS@Pt QDs, showing an enhancement of 300%, probably due to the spectral overlap between the Ag plasmonic band and the QD absorption, discussed in the previous sections, which may lead to absorption enhancement via local field concentration and to some contributions from energy transfer. This remarkably enhanced performance was observed up to 4 h of irradiation on freshly prepared satellite materials. However, we also noticed that the photo-catalytic enhancement was lost if the samples containing the hole-scavenger Na_2_SO_3_ solution were not fresh from the day, leaving a few open questions regarding the stability of the system in operando conditions.

## 3. Conclusions

We have developed a multi-step approach for the fabrication of nano-hybrid satellite materials combining a plasmonic core of AgNPs with photo-catalytic CdS@Pt QDs, stabilised by a spacing layer of SiO_2_ between the two active components. The materials were characterised with a series of complementary imaging and spectroscopic techniques, allowing the determination of the local chemical composition at the nano-scale and demonstrating the specific structures obtained at each fabrication step. We were able to measure reliably the hydrogen evolution from freshly prepared nano-hybrid satellites, showing a significant enhancement of 300% higher efficiency for hydrogen evolution, compared to the constituting photo-active components. Despite some issues with in-operando stability, this work paves the way for future applications on plasmonically enhanced catalysis using nano-hybrid materials for hydrogen generation.

## 4. Materials and Methods

### 4.1. Chemicals

Cadmium oxide (CdO, 99.998%), 1-octadecene (ODE, technical grade, 90%), oleic acid (technical grade, 90%), ammonium hydroxide (NH_4_OH, 28% *w*/*v*), 2-(4-morpholinol)ethanesulfonic acid (MES, 99%) were purchased from Alfa Aesar. Hydrochloric acid (HCl, 37% *w*/*v*), nitric acid (HNO_3_, 70% *w*/*v*), hexane (HPLC grade), ethanol (EtOH, absolute 99%), chloroform (CHCl_3_, analytical grade) and sodium sulfite (Na_2_SO_3_, anhydrous) were purchased from Fisher Scientific. (3-aminopropyl) triethoxysilane (APTES, 98%) and 4-(4,6-dimethoxy-1,3,5-triazin-2-yl)-4-methylmorpholinium chloride (DMTMM, 97%) were purchased from Flurorochem (Derbyshire, UK). Tetraethyl orthosilicate (TEOS, 99%) and triethanolamine (TEA, 99%) were purchased from Merck (Southampton, UK). L-ascorbic acid (AA, 99%) was purchased from VWR. All remaining chemicals listed were purchased from Sigma Aldrich (Gillingham, UK): silver nitrate (AgNO_3_, 99%), trisodium citrate dihydrate (Na_3_Ct, 99%), tannic acid (ACS reagent), sulfur (99.5%), 3-mercaptopropionic acid (MPA, 99%), tetramethylammonium hydroxide (TMAH, 97%), chloroplatinic acid hexahydrate (H_2_PtCl_6_. 6H_2_O, BioXtra), and 4-methylmorpholine (NMM, 99%). All chemicals were used as received without further purification. Deionised water (DI) (Thermo Scientific Barnstead Smart2Pure, Loughborough, UK) with a resistivity of 18 MΩ·cm was used throughout all experiments.

### 4.2. Synthesis of Silver Nanoparticles (AgNPs)

AgNPs were synthesised using a seed mediated approach adapted from Bastus et al. [22]. All glassware were first cleaned with aqua regia (3 HCl:1 HNO_3_) and then rinsed thoroughly a minimum of 3 times with deionised (DI) water prior to use. To synthesise the particles, in a 50 mL flask was added 44 mL of DI water, followed by the addition of 5 mL 50 mM sodium citrate (Na_3_Ct) and a 1 mL of 50 mM tannic acid to give a final volume of 50 mL. The solution was then heated under reflux at 110 °C until bubbling. Then, 1 mL of 25 mM silver nitrate (AgNO_3_) was added rapidly to the solution forming a yellow coloured solution of AgNPs. The solution was then left to cool to room temperature (RT) (approx. 25 °C) and then centrifuged at 12,000 Relative Centrifugal Force (RCF) for 30 min on a centrifuge Thermo Fisher Scientific. The supernatant was removed and the AgNPs were then re-dispersed back to their initial volume in 2 mM Na_3_Ct.

### 4.3. Preparation of AgNP@SiO_2_-NH_2_

The AgNPs were coated with silica (SiO_2_) following a modified *Stöber* process [23,24] and further functionalised with (3-aminopropyl) triethoxysilane (APTES) in a one-pot synthesis. In a typical synthesis, 3 mL of AgNPs stock solution were centrifuged at 12,000 RCF for 20 min. The supernatant was removed and the AgNPs were re-dispersed in 180 µL DI water. Then 62.5 µL of tetraethyl orthosilicate (TEOS) was added to the re-dispersed AgNPs and the mixed solution was subject to vortex for 2 min. The mixture was then injected rapidly into a 50 mL flask containing 3 mL EtOH and 250 µL of ammonium hydroxide (NH_4_OH) and stirred vigorously for 10 min. Next, 62.5 µL of APTES was added to the solution and stirred for a further 4 min. An excess (40 mL) of EtOH was then added to quench the reaction, thus preventing further hydrolysis. The solution was then collected and centrifuged at 12,000 RCF for 20 min and the now coated AgNPs were re-dispersed in DI water.

### 4.4. Synthesis of Cadmium Sulfide (CdS) Quantum Dots (QDs)

CdS QDs were fabricated following a procedure detailed by Yu et al. [20]. In a three-necked 50 mL flask (flask 1) fitted with a thermocouple and a septum, connected to a Schlenk line, 57 mg of cadmium oxide CdO, 20.25 mL 1-octadecene (ODE) and 2.15 mL oleic acid were mixed. In a separate two-necked flask (flask 2) fitted with a septum and connected to a Schlenk line, was added 12 mL of sulfur solution (38.5 mg sulfur in 60 mL ODE). Both solutions were then degassed for 30 min by purging with N_2_. Flask 1 was heated to 300 °C forming a clear colourless solution. Flask 1 was then cooled to 250 °C and allowed to stabilise. After cooling, 11.5 mL of the sulfur precursor in flask 2 was injected rapidly into flask 1, while maintaining rapid stirring. The temperature of the solution was then brought back up to 250 °C and held at that temperature for 10 min allowing the CdS nanocrystals to form. The heating jacket was then removed, and the solution was allowed to cool to RT. To purify the QDs, the reaction solution was divided into centrifuge tubes (~11 mL) and treated with a mixture of 10 mL hexane and 20 mL ethanol (EtOH), resulting in the formation of a yellow precipitate. The tubes were then cooled at 5 °C for 1 h and centrifuged at 15,000 RCF for 10 min and the clear supernatant was removed. The purification steps were repeated twice, and the final precipitate was re-dispersed back to the initial volume of the reaction solution in chloroform (CHCl_3_).

### 4.5. Preparation of 3-Mercaptopropionic Acid Functionalised CdS QDs

A sufficient quantity of CdS QD stock solution was used to give a final optical density (OD) of 1.5 at a wavelength of 415 nm, in a total volume of 10 mL CHCl_3_ (e.g., 1.725 mL OD 8.7 QDs and 8.275 mL CHCl_3_). To this solution 10 mL of DI water and 60 mg of 3-mercaptopropionic acid (MPA) were added. The pH of the solution was then adjusted to ~pH 9.0 via the drop wise addition of 0.5 M tetramethylammonium hydroxide (TMAH). The solution was then transferred to a separating funnel and shaken vigorously until the QDs moved into the upper aqueous layer. The biphasic mixture was then allowed to rest for 24 h prior to discarding the organic CHCl_3_ layer. The QDs were then centrifuged at 15,000 RCF for 20 min to separate them from any residual CHCl_3_. The aqueous layer containing the QDs was then transferred carefully into a 5 kDa Satorious Vivaspin^®^ (St. Neots, UK) ultrafiltration tube ultrafiltration tube and centrifuged at 8000 RCF for 60 min. The filtrate was removed and the QDs were re-dispersed in 15 mL DI water and centrifuged again at 8000 RCF for 60 min. The purification procedure was repeated once more and then the QDs were re-dispersed in 10 mL DI water giving a final OD of ~1.5 at a wavelength of 420 nm. The final solution was adjusted to pH 9.0 via the addition of 0.01 M TMAH prior to storing.

### 4.6. Photodeposition of Pt islands on CdS QDs

In a beaker, 10 mL of the 1.5 OD MPA functionalised QDs were added along with 651 mg of triethanolamine (TEOA), 65 mg L-ascorbic acid (AA), 11.67 mL DI water and 150 µL of 0.05 M chloroplatinic acid (H_2_PtCl_6_) [4,14,15]. The solution was stirred under continuous bubbling with argon for 30 min, followed by UV excitation at 365 nm for 20 min. The final solution was then decanted into a 5 kDa Vivaspin^®^ tube and centrifuged at 8000 RCF for 60 min. The filtrate was collected and the QDs were re-dispersed in 15 mL DI water and centrifuged again. The final material was re-dispersed in 21 mL of DI water leaving a solution with an OD ~0.5 at a wavelength of 420 nm.

### 4.7. Fabrication of the Nano-Hybrid Satellite Material

While stirring, 3 mL of the AgNP@SiO_2_-NH_2_ were added to a clean 50 mL flask along with the drop-wise addition of 300 µL of 4.73 mg mL^−1^ 4-(4,6-dimethoxy-1,3,5-triazin-2-yl)-4-methylmorpholinium chloride (DMTMM) and 300 µL 2.8 mg mL^−1^ of 4-methylmorpholine (NMM). Then, 3 mL of 0.5 OD CdS@Pt QDs were added to the reaction via a drop-wise addition. Next, 1.5 mL of 0.2 M pH 5.5 MES buffer were added to maintain a stable pH for the coupling reaction to occur. The final reaction solution was left to stir overnight at RT. The reaction solution was then centrifuged at 12000 RCF for 30 min to collect the final material and then re-dispersed in 3 mL DI water.

### 4.8. Ultraviolet-Visible (UV-Vis) Spectrophotometry

After fabrication, all the nanomaterials were characterised by UV-Vis spectroscopy in a quartz cuvette, with a path length of 1 cm. The solutions were diluted as required to avoid saturation of the detector. A Shimadzu (Buckinghamshire, UK) UV-2600 spectrophotometer with a resolution of 1 nm was used for all measurements.

### 4.9. Transmission Electron Microscopy (TEM) of Plasmonic Nanomaterials

All plasmonic nanomaterials, including the AgNPs, AgNP@SiO_2_-NH_2_ and the nanohybrid satellite material were characterised by TEM. The nanomaterials were first concentrated by centrifugation at 8000 RCF for 15 min, followed by drop-casting on a Pioloform^®^ coated 200 mesh copper (Cu) grid, forming a droplet. The droplet was then disturbed causing the droplet to collapse and the grid was left to dry. All samples were imaged using a FEI Tecnai G2 Spirit TEM (Cheshire, UK), with 120 keV. All images were processed using Fiji software [30].

### 4.10. Bright Field Scanning Transmission Electron Microscopy (BF-STEM), Coupled with Energy Disperse Spectroscopy (EDS)

All samples were characterised using a 200 kV JEOL 2100F Cs probe side aberration corrected analytical S/TEM (Tokyo, Japan) equipped with an EDAX Octane T Optima (Leicester, UK) windowless 60 mm^2^ SDD EDS detector. STEM-EDS was performed to map the chemical composition of the nano-hybrid satellite materials. Samples were prepared using a dilute solution of nanomaterials drop-cast on a holey-carbon coated Cu grid (200 mesh) and then vacuum dried using a vacuum desiccator. All images were post-processed using Fiji software.

### 4.11. X-Ray Photoelectron Spectroscopy (XPS)

XPS was used to characterise the AgNP@SiO_2_-NH_2_ and the nano-hybrid satellite material. After fabrication and purification, the solutions were drop-cast on either an aluminium coated silicon wafer or a silicon wafer and left to dry. These two substrates were used to eliminate the interference of silicon or aluminium from the respective substrate for accurate interpretation of both the silicon in the SiO_2_ layer and Pt islands, respectively. The XPS data was collected on a Kratos Axis Supra instrument (Hampshire, UK) using monochromatic Al Kα radiation (1486.7 eV, 225 W), with a low-energy electron flood source for charge compensation. Charge correction was done using the carbon 1s binding energy (284.8 eV). The XPS binding energies associated with the specific elemental peaks were identified using Kratos ESCApe software during analysis and further processed using CASA XPS software (Devon, UK) for display purposes.

### 4.12. Hydrogen Evolution Experiments

Hydrogen evolution experiments were carried out in a 5 cm long cylindric gas-tight glass reaction vessel, with a total volume of 18.2 mL. Two septum caps were fitted to the reaction vessel for deaerating the reaction solution and sampling the gas in the head space of the reaction vessel. In a typical experiment, the nanomaterials were prepared to a volume of 7 mL and an optical density (OD) of 0.3 over the path length of the 5 cm reaction vessel, at a wavelength of 420 nm or 430 nm for the CdS@Pt QDs and nano-hybrid satellite materials, respectively. In the 7 mL solution 1.4 mL of 0.1 M sodium sulfite (Na_2_SO_3_) was also added as a hole scavenger, leaving 11.2 mL of headspace. The solution was deaerated under magnetic stirring and bubbled with argon for 30 min and a sample of gas from the headspace was then collected and analysed using GC, to check for leaks and form a baseline prior to illumination. The sample was then illuminated with a 450 W FL-1039 Xe source (Horiba Scientific, Northampton, UK), fitted with a cooled IR filter to minimise heating of the sample. The lamp light was further attenuated using a band-pass filter (430 ± 70 nm). The power output of the lamp reaching the sample was measured using a thermal power sensor (Thorlabs S302C, Exeter, UK) to ensure equal incident power of 50 mW/cm^2^ on the reactor vessel for each experiment. A 300 µl aliquot of the gas from the headspace was collected every hour for 4 h and analysed by gas chromatography (GC) (Scion Instruments 436-GC, Livingston, UK) fitted with a BRP 81025 column (Bruker, Coventry, UK) packed with 5 Å molecular sieves. Argon was used as the carrier gas. The amount of hydrogen produced during the experiment was quantified by a thermal conductivity detector (TCD). The GC system was pre-calibrated using a commercial gas mixture (Scientific and Technical Gases Ltd, Staffordshire, UK) which contained 1% ± 0.02% of hydrogen, oxygen, and nitrogen in argon.

### 4.13. Inductively Coupled Plasma Optical Emission Spectrophotometry (ICP-OES)

After the hydrogen evolution experiments, the samples were collected and diluted by a factor of 2 ready for analysis by ICP-OES to determine the concentration of Cd and Pt in the solution, allowing normalisation of hydrogen evolution reaction rates by the concentration of Pt in the solution. The samples were analysed using an Agilent 5110 ICP-OES spectrometer (Cheshire, UK).

### 4.14. Quantification of AgNP Size and SiO_2_ Outer Coating Thickness

TEM images of AgNPs, AgNP@SiO_2_-NH_2_ and nano-Hybrid Satellites were processed and quantified using a homemade macro for Fiji image analysis software [30]. The complete pipeline is publicly available and detailed in https://github.com/ioritzsb/particle-analysis. Prior to image processing, TEM images were scaled to physical units and denoised using a median filter. Then, we used Trainable Weka Segmentation (TWS) algorithm [31] plugin to manually label each distinct material component within our TEM images (i.e., AgNP core and SiO_2_ coating) and generate a machine learning model to get multiclass segmentation masks after classification of unseen pixels within new sets of images. Multiclass segmentation masks were approximated with an ellipse, following a direct least square fitting of ellipse proposed by Fitzgibbon et al. [32], enabling splitting of connected components [33] and subsequent quantification of AgNP diameter and SiO_2_ thickness. Probability density functions of AgNP diameter and SiO_2_ thickness were represented by calculating a Gaussian kernel-density estimate using seaborn [34] statistical data visualisation package for Python. Comparison between SiO_2_ coating thickness for 6 min (*n* = 44), 10 min (*n* = 361) and 12 min (*n* = 764) coating times was performed using Welch’s ANOVA test following Dunnet’s T3 post-hoc test.

## Figures and Tables

**Table 1:** Photocatalytic hydrogen evolution results for free CdS@Pt QDs and nano-hybrid satellite material (SiO_2_ layer 13 nm) following 4 h of irradiation.

H_2_ Evolution Sample	Reaction Rate *	Enhancement Factor
CdS@Pt QDs	40.4	300%
Nano-Hybrid Satellite	120.8	

* Rates were normalised by the mol of Pt in the reaction solution

## Data Availability

The data that support the findings of this study and all custom codes are available from the corresponding author upon reasonable request.

## Supplementary Materials

The following are available online at https://www.mdpi.com/article/10.3390/nano11061580/s1, Representative data for automated TEM-image segmentation and particle size quantification (Figures S1 and S2 and Table S1), X-ray Photo-electron Spectroscopy analysis (Figures S3 and S4) additional UV-Vis analysis (Figure S5), ICP-OES determinations (Table S2), and EDS data (Figures S6–S8 and Table S3).
