# Supplementary material for: A Novel Self-Assembly Strategy for the Fabrication of Nano-Hybrid Satellite Materials with Plasmonically Enhanced Catalytic Activity
Source: Nanomaterials (Basel). 2021 Jun 16;11(6):1580. doi: 10.3390/nano11061580 (PMC8233724; doi:10.3390/nano11061580)
Supplement: Supplementary file 1 [file nanomaterials-11-01580-s001.zip › nanomaterials-1259966-supplementary.pdf]

SUPPLEMENTARY MATERIALS

A Novel Self-Assembly Strategy for the Fabrication of Nano-Hybrid Satellite Materials with Plasmonically Enhanced Catalytic Activity

Gareth Morris [1,2], Ioritz Sorzabal-Bellido [1,†], Matthew Bilton [3], Karl Dawson [4], Fiona McBride [1], Rasmita Raval [1,*], Frank Jäckel [2,*] and Yuri A. Diaz Fernandez [1,*]

1 Surface Science Research Centre, Department of Chemistry, University of Liverpool, Liverpool L69 3BX, UK; G.Morris@liverpool.ac.uk (G.M.); ioritz.sorzabal-bellido@liverpool.ac.uk (I.S.-B.); fiona.mcbride@liverpool.ac.uk (F.M.)

2 Stephenson Institute of Renewable Energy and Department of Physics, University of Liverpool, Liverpool L69 3BX, UK

3 Albert Crewe Centre for Electron Microscopy, University of Liverpool, Liverpool L69 3BX, UK; M.W.Bilton@liverpool.ac.uk

4 Department of Mechanical, Materials and Aerospace Engineering, School of Engineering, University of Liverpool, Liverpool L69 3BX, UK; K.Dawson@liverpool.ac.uk

* Correspondence: yuridiaz@liverpool.ac.uk (Y.A.D.F); fjaeckel@liverpool.ac.uk (F.J.); R.Raval@liverpool.ac.uk (R.R.)

† Current address: IDISNA, Ciberonc and Solid Tumors and Biomarkers Program, Center for Applied Medical Research, University of Navarra, 31009 Pamplona, Spain

TEM image processing pipeline for quantification of size distribution

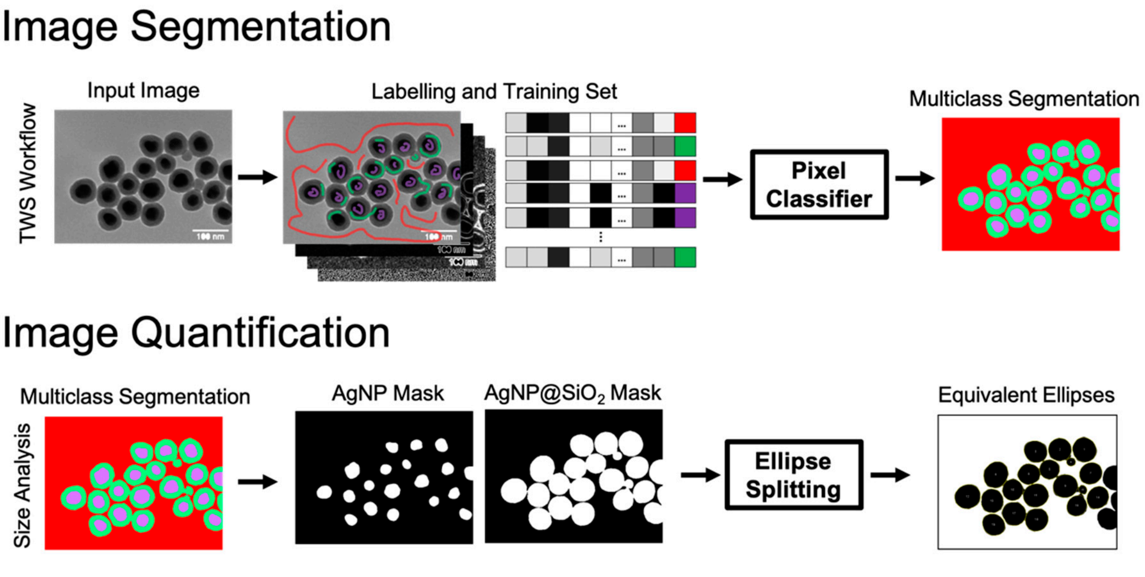

Figure S1. TEM image processing pipeline used in this work for quantification of nanomaterial size distribution, available in https://github.com/ioritzsb/particle-analysis. Image analysis pipeline is composed of two steps: first, TEM images are segmented using Trainable Weka Segmentation (TWS) machine learning plugin for FIJI. Then, generated multiclass segmentation masks are used to create binary masks of AgNP and AgNP@SiO_2 to subsequently obtain AgNP size and SiO_2 coating thickness by fitting of equivalent ellipses on foreground image features.

TEM images and segmentation masks for AgNPs, AgNP@SiO_2 and nano-Hybrid Satellite

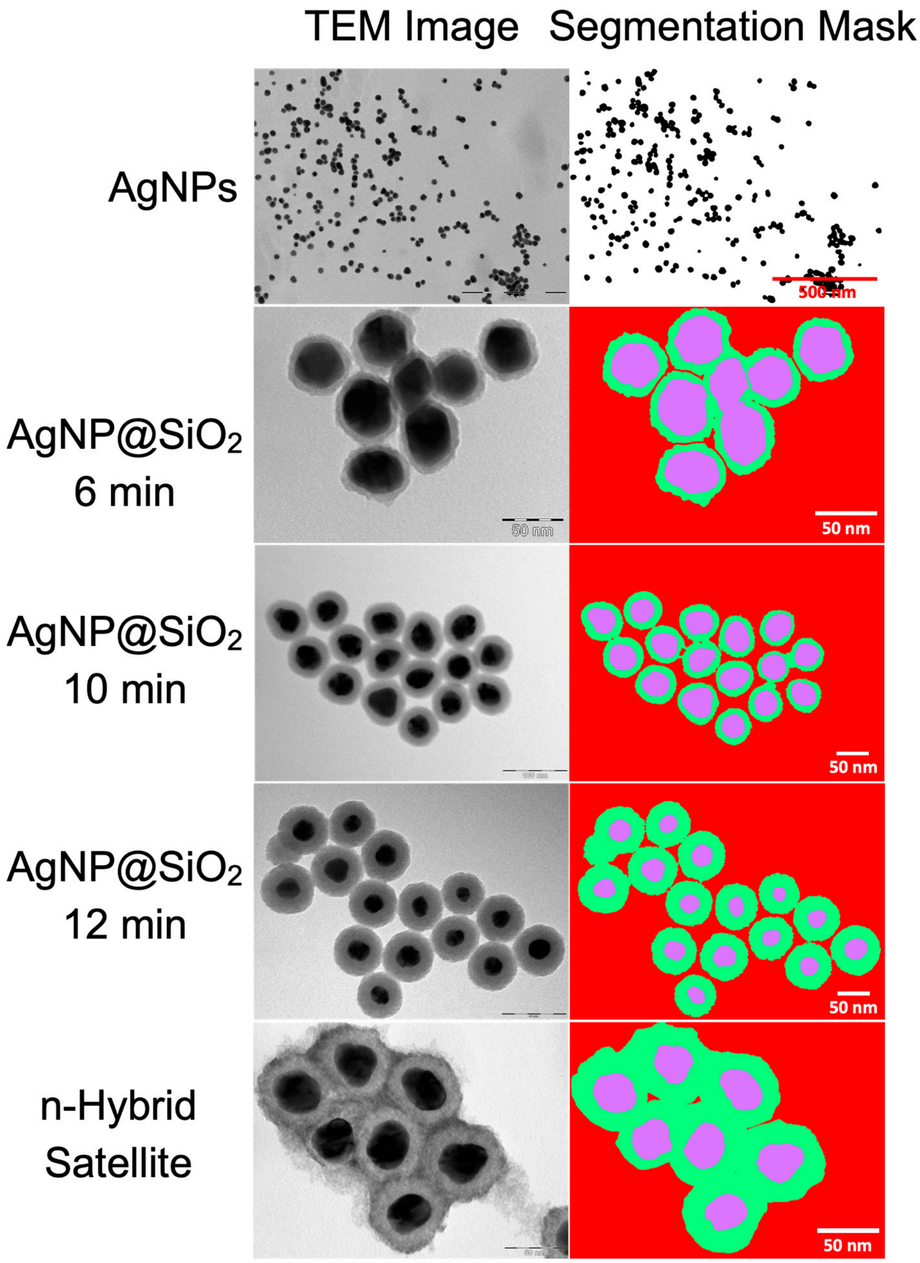

Figure S2. Raw TEM images (left) and segmentation masks (right) of AgNPs, AgNP@SiO_2 at different SiO_2 coating times and nano-Hybrid Satellites. Segmentation masks were obtained following the TEM image processing pipeline illustrated in Figure SI1.

AgNP diameter and SiO_2 thickness distribution parameters

Table S1. Location and dispersion parameters of AgNP diameter and SiO_2 coating thickness on fabricated nanomaterials. Std, Q1 and Q3 indicate standard deviation, 1st quartile, and 3rd quartile, respectively.

	**Diameter, nm**	**SiO_2 Thickness, nm**			
	**AgNPs**	**AgNP@SiO_2-NH_2**			**n-Hybrid Satellite**
**SiO_2 Coating Time**	**None**	**6 Min**	**10 Min**	**12 Min**	**10 Min**
Mean	41.7	4.7	9.6	24.2	16.0
Stdev	9.1	1.4	4.1	4.6	3.4
Median	42.6	4.9	9.3	24.7	15.5
Q1	37.6	3.8	7.9	22.1	13.3
Q3	46.4	5.6	11.0	26.4	17.6

X-ray Photoelectron Spectroscopy (XPS) analysis

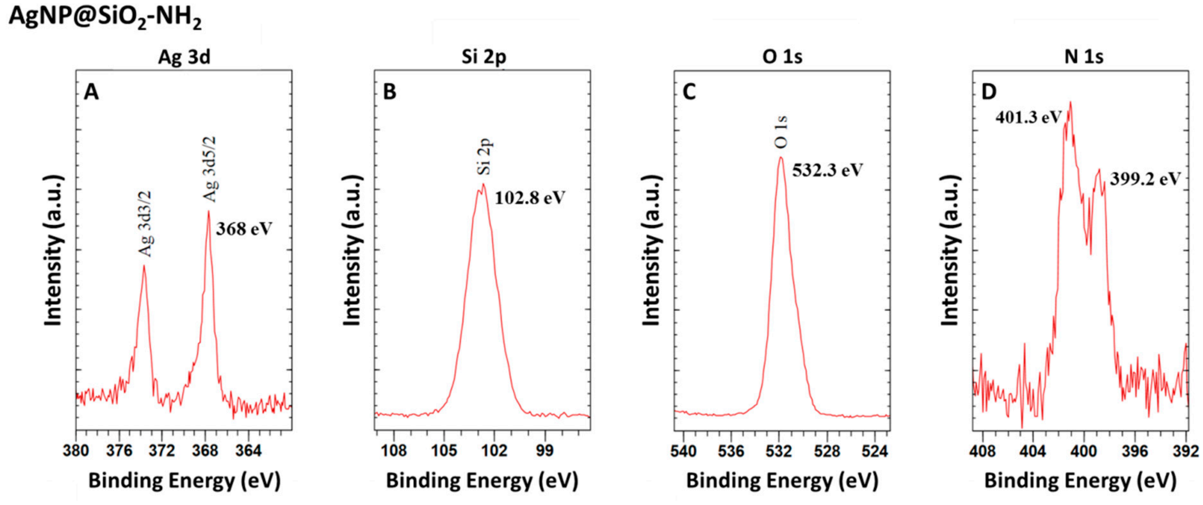

Figure S3. XPS spectra of the intermediate material AgNP@SiO_2-NH_2, showing the presence of the key elements Ag, Si, O and N, on the sample.

Analysis of the optical properties for CdS@Pt QDs and AgNP@SiO_2-NH_2

The individual optical properties of the precursor nanomaterials are presented in Figure SI5A. We note that there are specific regions of the spectrum where only one of the two precursors contributes significantly to the total extinction. Above 450 nm only AgNP@SiO_2-NH_2 contributes to the optical density, while below 350 nm the optical properties are dominated by CdS@Pt QDs. We attempted to use these mutually exclusive regions to normalise each of the individual spectra for the components to reconstruct the spectra of the nano-hybrid satellite system, but the resulting theoretical optical density from this lineal combination did not match the experimental spectrum of the satellites (Figure SI5B).

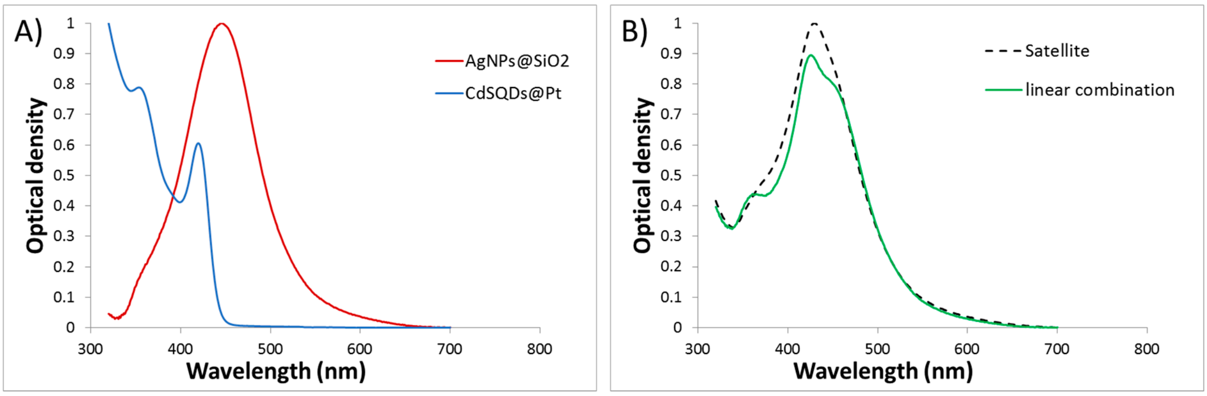

Figure S4. Optical properties of the materials A) Experimental normalised extinction spectra for the precursor nanomaterials AgNP@SiO_2-NH_2 and CdS@Pt QDs; B) Experimental normalised extinction spectrum for the nano-hybrid satellite material superimposed with the theoretical spectrum obtained by the best-matched linear combination of the individual spectra from the precursor nanomaterials.

X-ray Photoelectron Spectroscopy (XPS) analysis

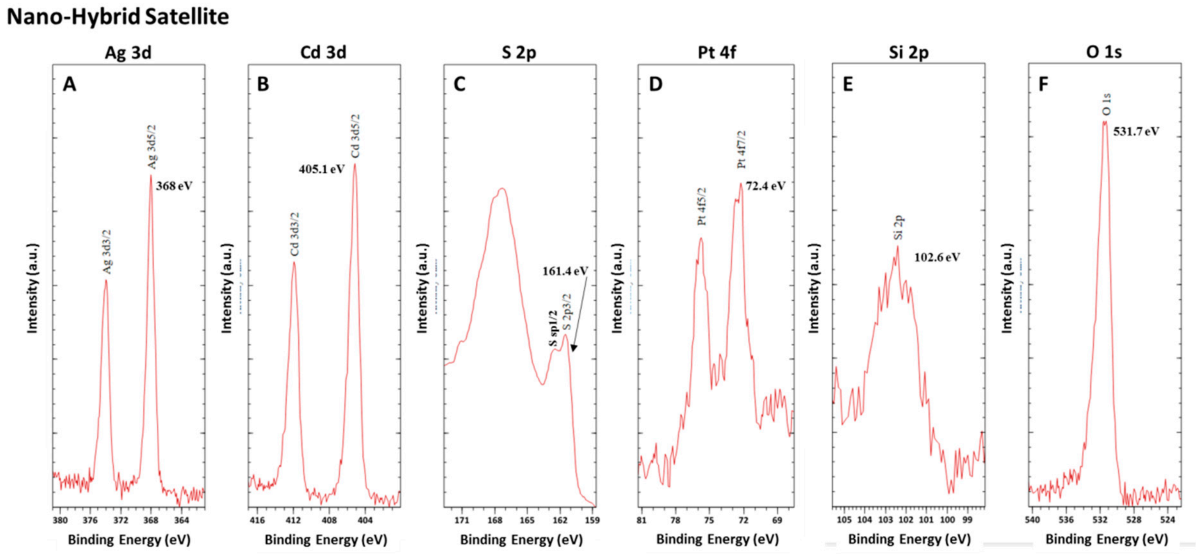

Figure S5. XPS spectra of the final nano-hybrid satellite material, showing the presence of the key elements Ag, Si, and O from the plasmonic core, and Cd, S, and Pt, from the satellite QDs.

ICP-OES determination

Table S2. Total concentration of Cd and Pt in the original reaction solutions, corrected by any dilution factor needed for ICP-OES analysis.

H_2 Evolution Sample	Cd (ppm)	Pt (ppm)
CdS@Pt QDs	17.6	0.2
Nano-Hybrid Satellite	15.3	0.3

These concentration values for Pt were used to calculate the normalised reactions rates, using equations S1 and S2.

$$\textbf{\textit{Reaction rate}}_{H_2/Pt} = \frac{\textit{Moles } H_2}{\textit{Moles Pt} \cdot \Delta t} \qquad \text{(S1)}$$

$$\textit{Moles } H_2 = \textit{Mol}\% \times \left(\frac{\textit{Headspace Volume}}{\textit{Molar Volume of Ideal gas}}\right) \qquad \text{(S2)}$$

Additional Scanning Transmission Electron Microscopy and Energy Dispersed Spectroscopy (STEM-EDS) data.

Table S3. EDS Energies (keV) for key elements (source: https://www.edax.com/)

	Si	S	Ag	Cd
Kα	1.740	2.307	22.116	23.175
Lα	0.092	0.149	2.964	3.133

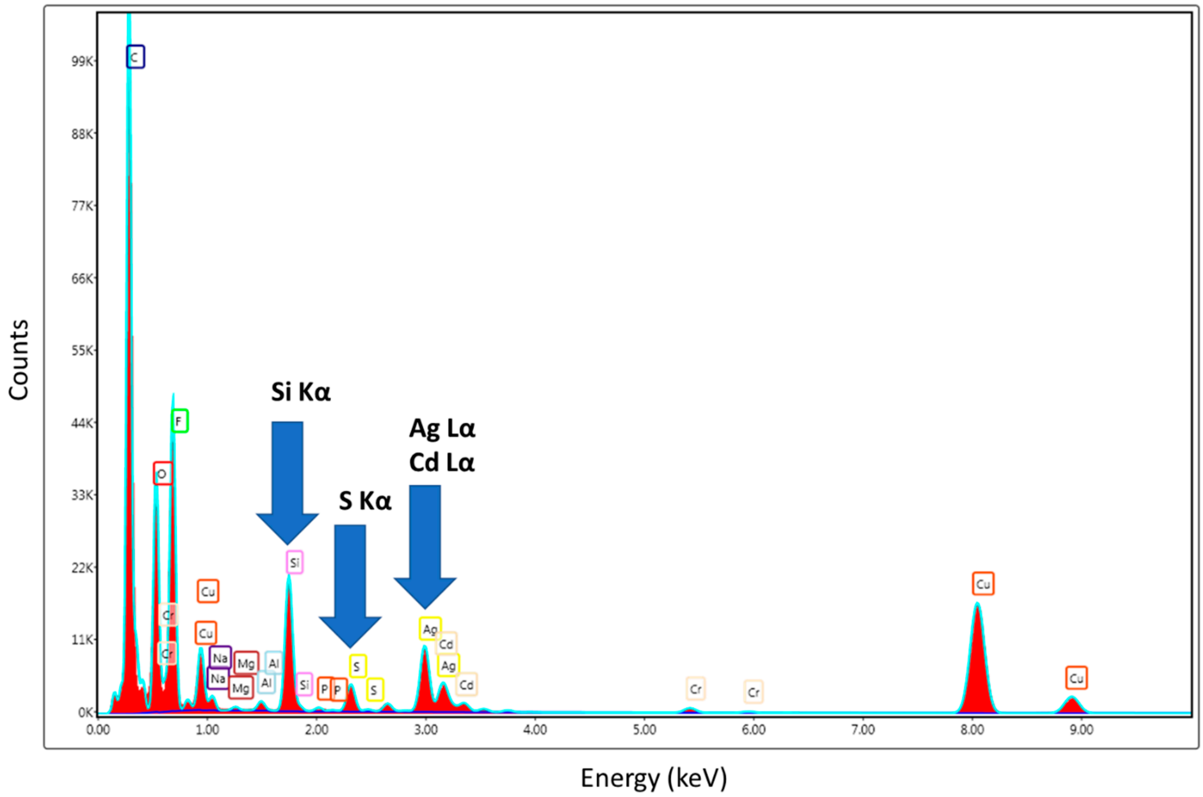

Figure S6. Representative STEM-EDS spectrum of the satellite material in the low energy range, showing the presence of Si, S, Ag, and Cd. Note the energy overlap for Lα peaks of Ag and Cd that precluded the resolution of chemical spatial distributions for these two elements (as shown in main text Figure 7). Additional EDS peaks coming from the TEM copper grid and instrument background were also detected.

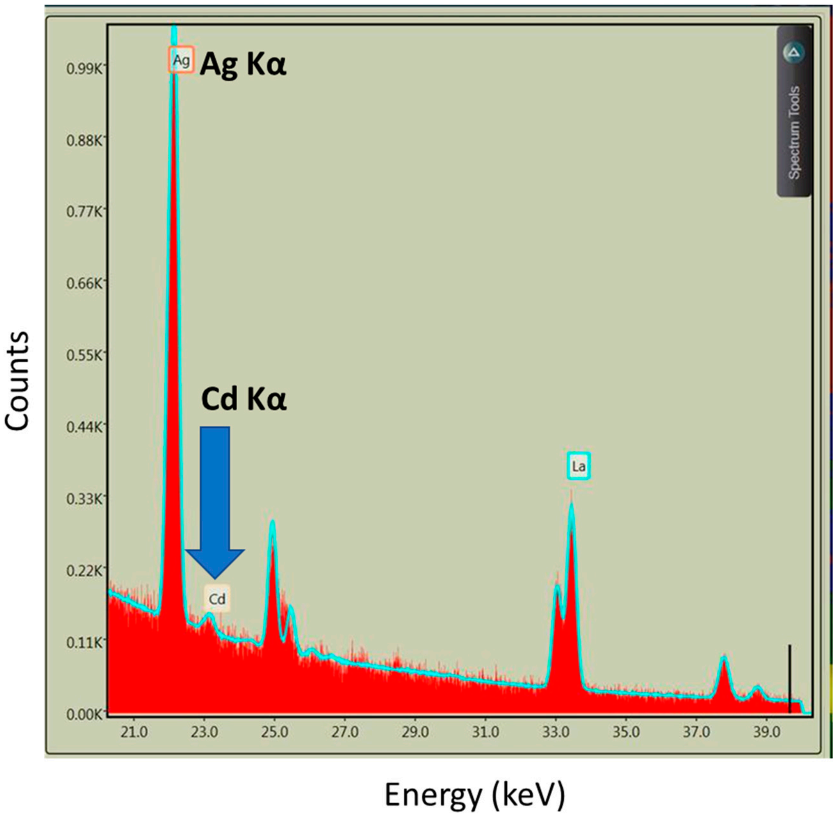

Figure S7. Representative STEM-EDS spectrum of the satellite material in the high energy range showing distinctively the presence of Ag and Cd in the sample. Although better energy separation was observed for the Kα peaks of Ag and Cd, the relatively low intensities of these peaks led to poorer quality maps for elemental distribution (See Figure SI7C). Additional EDS peaks coming from the TEM copper grid and instrument background were also detected.

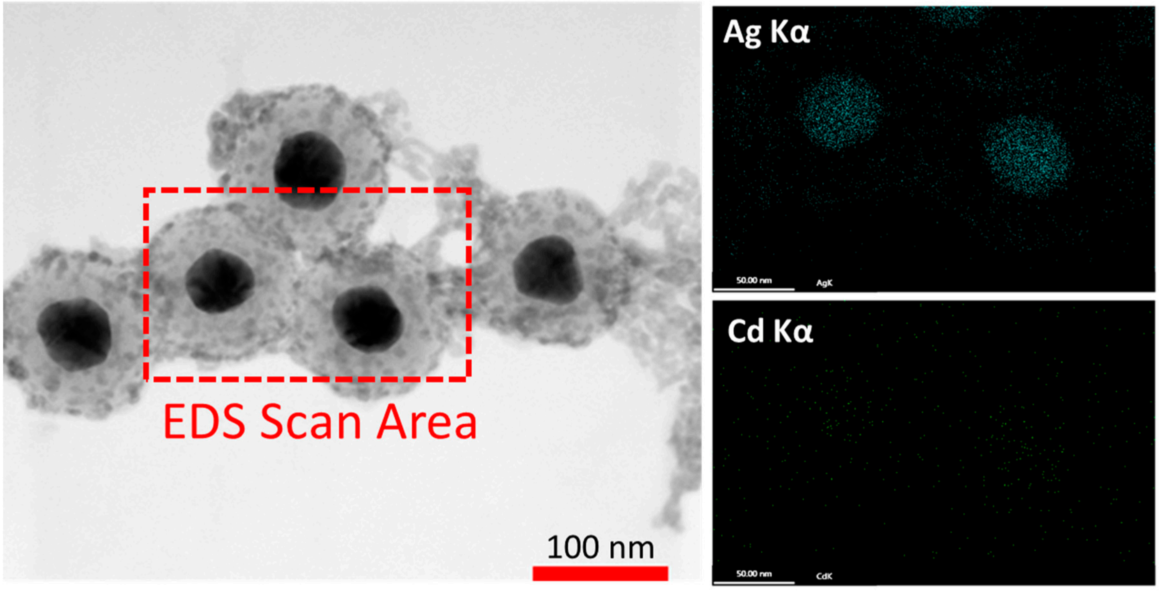

Figure S8. STEM-EDS mapping of Kα peaks for Ag and Cd, showing the poor quality for Cd Kα map due to the relatively lower intensity of this peak. These images are complementary to Figure 7 in the main text.
